# Epigenetic alterations are associated with monocyte immune dysfunctions in HIV-1 infection

**DOI:** 10.1038/s41598-018-23841-1

**Published:** 2018-04-03

**Authors:** Milena S. Espíndola, Luana S. Soares, Leonardo J. Galvão-Lima, Fabiana A. Zambuzi, Maira C. Cacemiro, Verônica S. Brauer, Cleni M. Marzocchi-Machado, Matheus de Souza Gomes, Laurence R. Amaral, Olindo A. Martins-Filho, Valdes R. Bollela, Fabiani G. Frantz

**Affiliations:** 1 Faculdade de Ciencias Farmaceuticas de Ribeirao Preto, Universidade de Sao Paulo, Ribeirao Preto, SP Brazil; 2 Laboratorio de Bioinformatica e Analises Moleculares – INGEB/FACOM, Universidade Federal de Uberlandia, Patos de Minas, MG Brazil; 3 Laboratorio de Biomarcadores para Diagnostico e Monitoramento, Centro de Pesquisas Rene Rachou, FIOCRUZ, Belo Horizonte, MG Brazil; 4 Faculdade de Medicina de Ribeirao Preto, Universidade de Sao Paulo, Ribeirao Preto, SP Brazil

## Abstract

Monocytes are key cells in the immune dysregulation observed during human immunodeficiency virus (HIV) infection. The events that take place specifically in monocytes may contribute to the systemic immune dysfunction characterized by excessive immune activation in infected individuals, which directly correlates with pathogenesis and progression of the disease. Here, we investigated the immune dysfunction in monocytes from untreated and treated HIV + patients and associated these findings with epigenetic changes. Monocytes from HIV patients showed dysfunctional ability of phagocytosis and killing, and exhibited dysregulated cytokines and reactive oxygen species production after *M. tuberculosis* challenge *in vitro*. In addition, we showed that the expression of enzymes responsible for epigenetic changes was altered during HIV infection and was more prominent in patients that had high levels of soluble CD163 (sCD163), a newly identified plasmatic HIV progression biomarker. Among the enzymes, histone acetyltransferase 1 (HAT1) was the best epigenetic biomarker correlated with HIV - sCD163 high patients. In conclusion, we confirmed that HIV impairs effector functions of monocytes and these alterations are associated with epigenetic changes that once identified could be used as targets in therapies aiming the reduction of the systemic activation state found in HIV patients.

## Introduction

Monocytes are one of the most important innate immune cells associated with immunopathogenesis in HIV infection, even though monocytes are poorly or not directly infected by HIV. The events that take place specifically in monocytes may contribute to the systemic immune dysfunction characterized by excessive immune activation in individuals infected with HIV-1, which correlates directly with the pathogenesis and progression of the disease^1–3^. Among newly described plasma biomarkers in HIV, plasma levels of the monocyte/macrophage activation marker, soluble CD163 (sCD163), the released form of the hemoglobin scavenger receptor in response to inflammatory signals, associate with clinical outcomes and predict all-cause mortality in HIV-1 infected individuals^4–8^. Additionally, high sCD163 levels correlate with neurocognitive impairment in HIV infection, postmortem brain pathology^9^, and predicts incident chronic lung, kidney and liver disease in HIV infection^10^.

Combination antiretroviral therapy (cART) is the current treatment of choice for HIV, it completely inhibits circulating viremia and partially improves health and restore CD4 + T cell counts. However, patients under therapy frequently experience the development of non-AIDS disorders including cardiovascular disease, kidney disease, liver disease, malignancy, and some neurological diseases^11^. Clinical trials aiming at reduction of chronic inflammation and the effects of cART in HIV + patients have been widely performed. Therapies focused in reducing microbial translocation^12–15^; co-pathogens excess^16^; systemic inflammation and the hypercoagulation state^17–20^ remain in tests. However, given the complexity and multiple factors associated with HIV infection, the use of certain therapies could reduce the immunological ability to control viral replication or activate compensatory pathways^1^.

Epigenetic mechanisms are defined by changes in gene expression without altering the DNA sequence. It includes several chromatin alterations as methylation, phosphorylation, acetylation and ubiquitination of histones or direct DNA methylation performed by specialized enzymes^21^. These alterations in diseases can modify host immune response by altering gene expression profile and further protein production. In the case of HIV, epigenetics are involved in regulation of viral latency^22^ and viral replication^23^. How HIV may epigenetically alter the response of innate immune cells is still unknown. Therapies aiming at modulating epigenetic enzymes are innovative strategies in the regulation not only of viral replication but more importantly the immune response against the virus and opportunistic infections, such as tuberculosis, common in these patients. Ongoing clinical trials against various types of cancer^24^ and inflammatory diseases^25^ show promising results in this field. In HIV infection, therapies focused on eliminating viral latency, using Vorinostat, an HDAC inhibitor are also being tested^26^. Once elucidated, epigenetic biomarkers could be used as targets in HIV gene therapy, aiming to reduce systemic immune activation, the central cause of morbidity and mortality in HIV-infected patients in the cART era.

In this sense, the aim of this work was to characterize monocytes from HIV + patients and correlate the immune dysfunction and progression with epigenetic alterations in these cells. Here, we show that HIV impairs the function of monocytes by reducing phagocytosis and killing and inducing dysregulated production of pro-inflammatory mediators when these cells are challenged with *M. tuberculosis* (Mtb). Epigenetic biosignature of monocytes from HIV patients display a more activated state, that is stronger when patients are segregated according to progression.

## Results

### Functional ability of monocytes is impaired in HIV + cells and is partially recovered after cART

**Figure 1:**
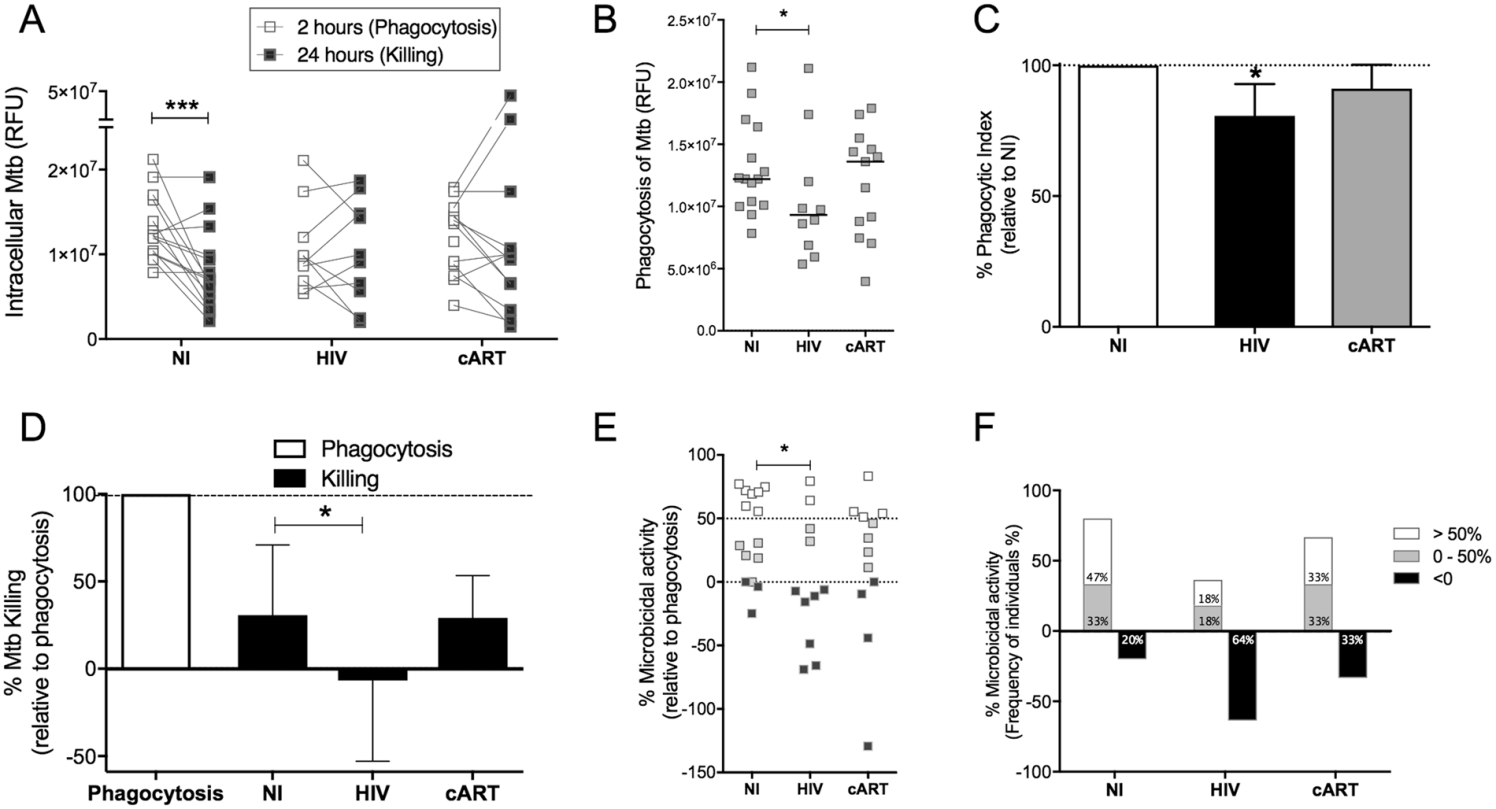
Functional activity of monocytes is reduced in HIV + patients and partially recovered after cART. Phagocytosis and microbicidal ability of monocytes from non-infected individuals (NI), untreated HIV + patients (HIV) or HIV + patients under cART (cART) were evaluated after Mtb infection *in vitro*. CD14 + monocytes were magnetically sorted and infected with Mtb (MOI 5) *in vitro*. Phagocytosis, evaluated after 2 hours of infection and the microbicidal activity, evaluated after 24 hours of infection, were performed separately using resazurin reduction assay by intracellular Mtb after 24 hours of incubation following the infection times. (**A**) Intensity of intracellular Mtb fluorescence after 2 hours (Phagocytosis) and 24 hours of infection (Killing). (**B**) Intracellular Mtb fluorescence intensity and (**C**) Phagocytic index after 2 hours of phagocytosis. (**D**) Percentage of microbicidal activity of monocytes compared to phagocytosis. (**E**) Percentage of microbicidal activity segregated according to the extent of bacillus elimination. (**E**) Frequency of individuals with >50%, 0–50% or <0% of Mtb killing. In graphs A and B the data is expressed in RFU (Relative fluorescence unit), and in B the line represents the median of the group. *P < 0.05; ***P < 0.001 versus NI. In Graph D results are expressed as median ± Interquartile Interval. *p < 0.05.

In order to assess whether HIV infection would interfere with the functional features of monocytes, we evaluated the phagocytic and microbicidal ability of these cells from non-infected controls (NI), untreated HIV + patients (HIV) and HIV + patients under cART (cART) after *in vitro* challenge with *M. tuberculosis*. Phagocytosis and killing of Mtb were assessed 2 and 24 h after infection, respectively. We observed significant reduction of intracellular Mtb after 24 h on monocytes from NI controls but not from HIV patients regardless treatment (Fig. 1A). Phagocytosis was impaired on monocytes from untreated patients, which was reversed upon cART treatment compared to NI (Fig. 1B and C).

Untreated HIV + patients showed dysfunctional ability of killing or controlling bacterial growth on monocytes since the median percentage of Mtb killing is below zero, indicating bacillus growth over its containment. On the other hand, monocytes from HIV + patients under cART demonstrated similar microbicidal capacity to NI individuals (Fig. 1D). Given the heterogeneity of the studied clinical groups, we individually analyzed the ability of monocytes to eliminate Mtb and classified individuals according the degree of microbicidal activity in: above 50%; between 0–50% and below zero (Fig. 1E). We found that among NI individuals, 47% had microbicidal activity above 50%; 33% between 0–50% and only 20% of individuals in this group were not able to control the growth of tuberculosis bacilli. On the opposite side, among untreated HIV + patients, only 18% of the subjects could eliminate more than 50% of the bacteria; 18% were between 0–50% and 64% of individuals in this group were unable to control the growth of Mtb. Among the patients who used cART, 33% showed microbicidal activity above 50%, 33% between 0–50% and 33% below zero (Fig. 1F).

### Monocytes from HIV + patients show dysregulated responsiveness to Mtb stimulus

**Figure 2:**
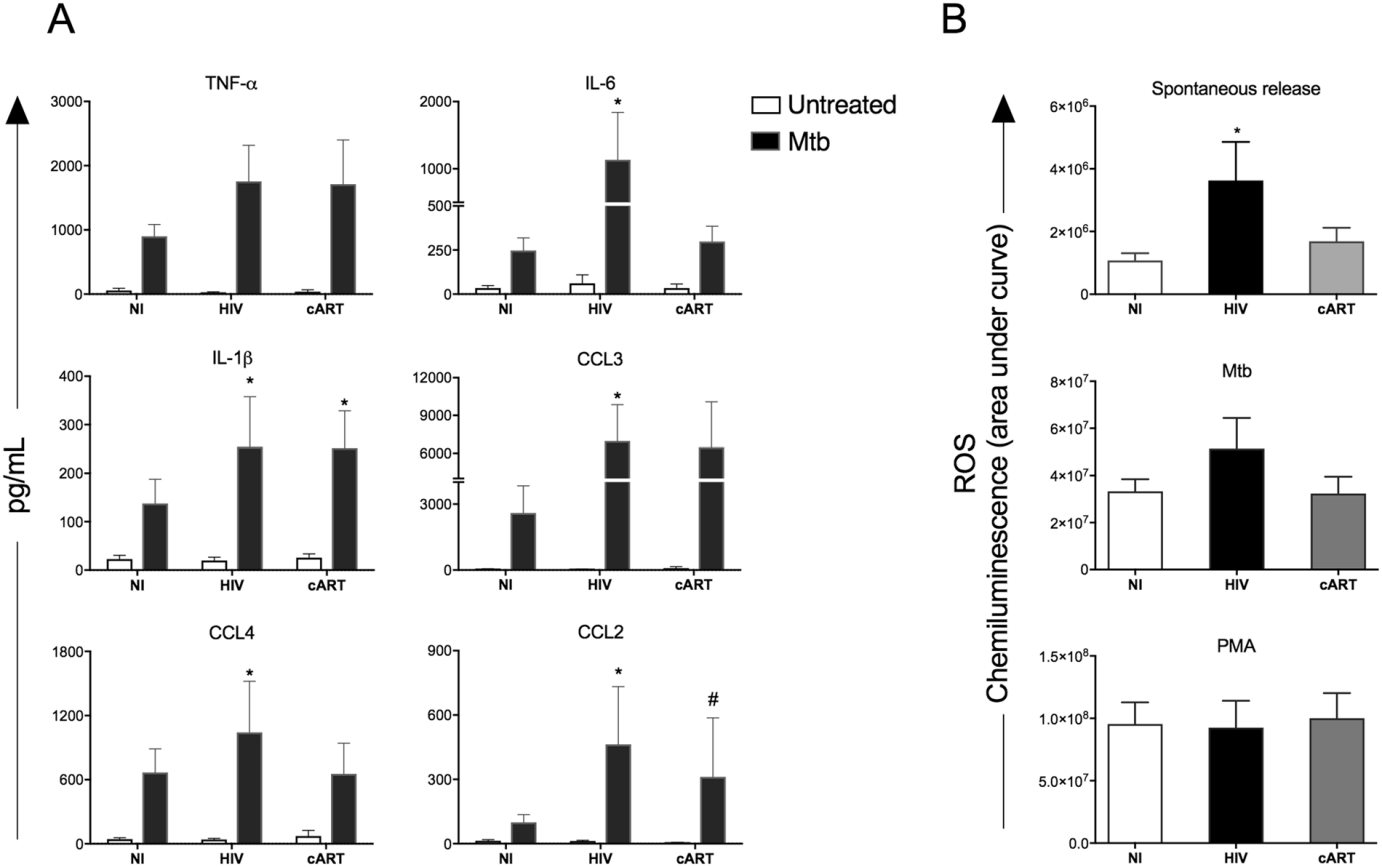
Monocytes from untreated HIV + patients show exacerbated production of immune mediators after Mtb stimulation. (**A**) Cytokines and chemokines were evaluated in monocytes from non-infected individuals (NI), untreated HIV + patients (HIV) or HIV + patients under cART (cART) 24 h after Mtb infection (MOI 5) *in vitro*. The cytokines and chemokines TNF-α, IL-6, IL-1β, CCL2, CCL3 and CCL4 were quantified using magnetic beads from MILLIPLEX MAP kits® Human Cytokine/Chemokine (Millipore). The result is expressed as mean ± SEM of the cytokine production of unstimulated or stimulated monocytes with Mtb. *P < 0.05 versus NI; ^#^P < 0.05 versus HIV. (**B**) Reactive oxygen species were evaluated in monocytes from non-infected individuals (NI), untreated HIV + patients (HIV) or HIV + patients under cART (cART). Luminol-based assay was performed in unstimulated, heat-killed Mtb (MOI 5) or PMA (10^−7^M) stimulated cells. Immediately after stimulation, chemiluminescence was assessed in a luminometer for 2 hours of kinetics. The results are expressed as area under chemiluminescence curve. *p < 0.05 vs NI.

Among the major cytokines and chemokines produced by monocytes, IL-6, IL-1β, CCL2, CCL3 and CCL4 were differentially produced by cells from untreated HIV + patients after Mtb stimulus compared to NI individuals. IL-1β was also increased in monocytes from cART treated patients, compared with NI individuals. Oppositely, the production of CCL2 was reduced in Mtb stimulated cells from patients under cART compared to the ones from untreated HIV + patients. The levels of TNF-α and CCL3 were similar in monocytes from untreated and treated HIV + patients, while IL-6 and CCL4 levels were similar to the levels of cells from NI individuals (Fig. 2A).

Accordingly, in addition to cytokines production, we assessed the ability of these cells to produce ROS spontaneously, after stimulation with heat-killed Mtb or with PMA, as a positive control. There was an increase in the spontaneous production of ROS by cells from untreated HIV + patients, compared to NI individuals. Moreover, the use of cART reduced spontaneous ROS release to the levels of NI individuals. After Mtb stimulation there was an increased production of ROS by monocytes of all clinical groups, although cells from untreated HIV + individuals showed a stronger response to Mtb. As expected, stimulation with PMA induced the maximum release of ROS regardless the clinical group (Fig. 2B).

### Epigenetic biosignature of HIV + patients show altered frequencies of enzymes associated to activation and repression mechanisms

To evaluate whether the dysfunctional immune response of monocytes from untreated HIV + patients and HIV + patients under cART correlate to epigenetic alterations, which are changes in the dynamics of DNA accessibility and gene transcription, we performed analysis of global DNA methylation and frequency of the enzymes responsible for epigenetic changes, that include activation or repression of gene transcription.

The pattern of global DNA methylation was approximately 17% in NI individuals. In contrast, untreated HIV + patients showed a significant reduction in DNA methylation compared to NI individuals, averaging 9%, which corresponds to 48% less methylated DNA than NI group. The use of cART restored methylation pattern to the levels of NI individuals (Fig. S1).

**Figure 3:**
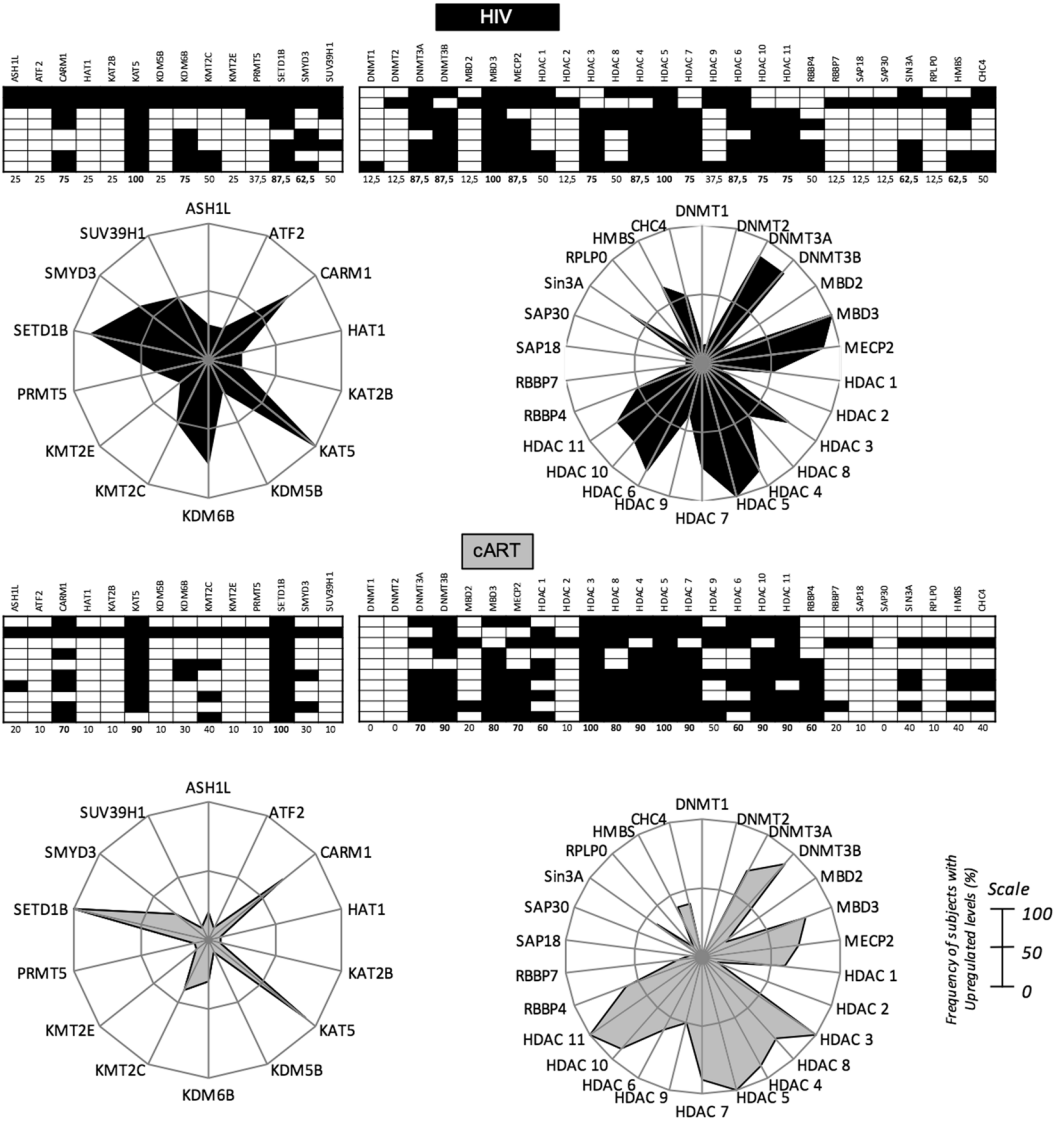
Epigenetic biosignature of monocytes from untreated or treated HIV + patients. Gene expression analysis of the enzymes responsible for activation or repression of transcription was used for categorical classification of untreated HIV + patients or those under cART according to the status of expression, upregulation and downregulation, of a given gene compared to NI individuals. Black and white diagrams represent upregulated and downregulated genes, respectively. Each lane represents a gene and each block represents the status of expression compared to NI. Numbers below each lane represent the frequency of patients with high gene expression of each enzyme evaluated. Radar charts summarize the percentage of patients with high gene expression of each studied clinical group. When the frequency was greater than 50% (on a 0–100% scale), the result was highlighted. NI (n = 10); HIV (n = 8); cART (n = 10).

The changes in the expression dynamics of the enzymes, especially the ones related to changes in methylation and acetylation, were investigated according to their frequencies. We have analyzed gene expression of 26 enzymes and proteins associated with repression and 14 enzymes and proteins associated with activation of transcription in monocytes from non-infected individuals, untreated HIV + or cART treated patients. As an initial comprehensive investigation, we evaluated the epigenetic biosignature from the frequency of patients that had upregulated genes compared to NI. For that matter, we used categorical analysis strategies, in which the number of subjects with increased or decreased gene expression levels was compared to NI individuals and compiled in a black-and-white scale diagram to determine the frequency of individuals with increased gene expression of each targeted gene (Fig. 3). By using radar charts for comparison among clinical groups, we determined significant differences in the overall gene expression profiles when the frequency of upregulated genes was greater than 50%.

Regarding activation of gene transcription, we found that HIV infection induced increase in the frequency of patients with high expression of CARM1, KAT5, KDM6B, SET1B and SMYD3. Upon the use of cART, there was a reduction in expression of KDM6B and SMYD3 when compared to untreated HIV + patients (Fig. 3, left charts).

On the other hand, considering the enzymes involved in repress gene expression mechanisms, we observed an increased frequency of individuals with high expression of the DNA-methyltransferases DNMT3A and DNMT3B while DNMT1 and DNMT2 were downregulated among untreated HIV + patients, compared to NI individuals. MBD3 and MECP2, which are Methyl-CpG-binding proteins, were upregulated in most HIV + patients. We also analyzed the histone deacetylases class I, IIA, IIB and IV and observed upregulation of HDAC3, HDAC4, HDAC5, HDAC7, HDAC6, HDAC10 and HDAC11 in more than 50% of untreated HIV patients. Among the enzymes that act to form complexes with other proteins, here called accessory proteins, the expression profile of RBB4 and RBB7, SAP18, SAP30, SIN3A, RPLP0, HMBS and CHC4 was investigated. We found that SIN3A and HMBS were also increased in most untreated HIV + patients (Fig. 3, right upper chart).

When compared to untreated, those under cART showed similar frequency pattern to individuals with upregulated genes, and additionally showed increased frequencies of individuals with upregulation of HDAC1 and HDAC8 genes and downregulation of HDAC6, SIN3A and HMBS that were not observed in the untreated HIV + group (Fig. 3, right bottom chart).

### Heat map analysis reveals segregation of epigenetic activation and repression mechanisms between non-infected and HIV + patients

We next addressed how the global gene expression of enzymes that perform epigenetic activation or repression in monocytes from NI or HIV + patients would cluster in a heat map. Genes responsible for activation (in blue) or repression of transcription (in red) defined opposite clusters on the heat map. Moreover, monocytes from NI patients tended to cluster together between HIV and treated patients. Interestingly, enzymes responsible for shutting down gene transcription are upregulated in NI cells, evidencing a well-controlled mechanism of gene regulation. In opposition, cells from HIV patients regardless of treatment, show an overall reduction of repression mechanisms. Two untreated HIV patients (HIV22 and HIV20) showed a different pattern from all others, characterized by notorious upregulation of genes responsible of activation mechanisms.

**Figure 4:**
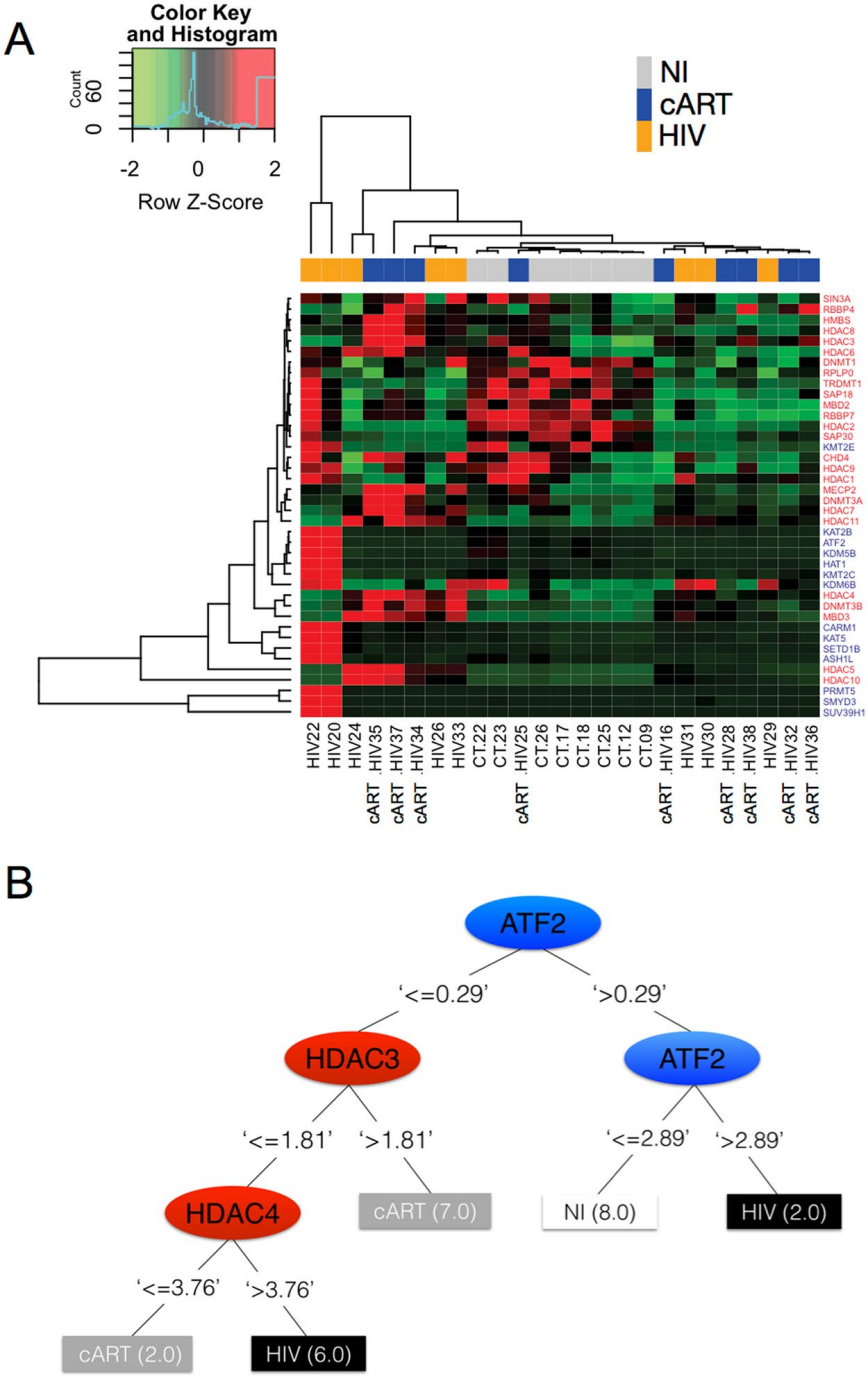
Heat map of enzymes responsible for epigenetic changes defines a segregation profile upon activation or repression of transcription and between the NI and HIV groups. (**A**) Gene expression of enzymes responsible for activation or repression of transcription were used to calculate the Z-score for each gene produced by subjects individually from NI, HIV or cART groups, for further generation of a heat map. Row Z-score scaled from −2 to + 2 and is illustrated as green, black, and red colors. (**B**) The values of the gene expression were used for the decision tree dataset construction. The decision tree was generated considering the three groups separately. The numbers beside group names indicate the quantity of subjects ranked on each pathway.

By using this strategy, it was not possible to distinguish untreated from treated patients, however based on this analysis, we constructed a decision tree in order to classify the key epigenetic biomarkers able to differentiate NI, untreated HIV and HIV treated groups. The most important attribute for the decision tree was the activation enzyme ATF2, followed by HDAC3 and ATF2. When ATF2 was higher than 0.29 and followed by ATF2 again it was possible to differentiate between all NI and the group of 2 untreated HIV patients. On the other hand, when ATF2 was bellow 0.29 and followed by HDAC3 and HDAC4 it was possible to distinguish treated from untreated HIV patients (Fig. 4B).

### Soluble CD163 levels, a plasma biomarker of disease progression, outline the expression of epigenetic activation or repression related enzymes on untreated HIV + patients

To determine whether progression would play a role in the expression pattern of these enzymes on monocytes, we used plasmatic sCD163 levels, a marker of monocyte activation that predicts mortality in HIV-1 infected individuals^7^, as a putative biomarker of progression. High levels of sCD163 directly correlate with well-known clinical progression outcomes as low CD4 T cell counts and CD4/CD8 ratio; high viremia and plasmatic IP-10 (Fig. S2). We have previously associated high plasmatic sCD163 to a strong inflammatory profile, that correlates with a poor prognosis, to separate putative slow from putative rapid progressors, as reported previously by our group^27^. Here we segregated untreated HIV + patients into sCD163 low and sCD163 high (Fig. S3) to correlate monocytes epigenetic status with cell activation.

**Figure 5:**
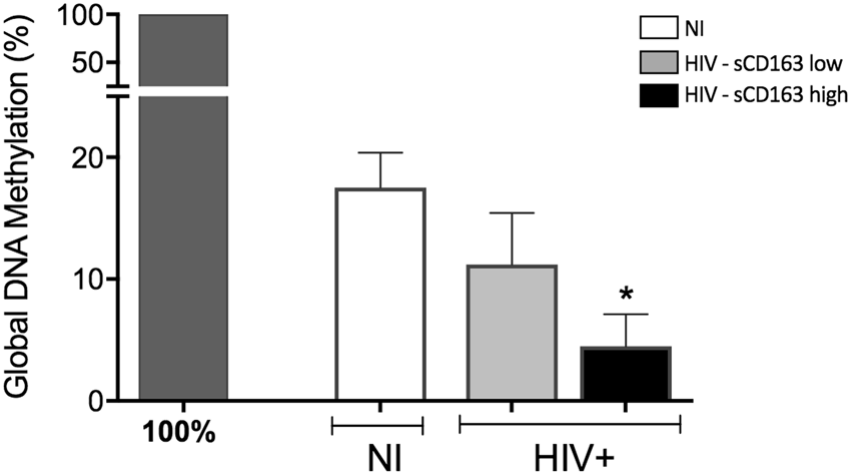
Percentage of global methylation of genomic DNA differs according to the levels of sCD163 in untreated HIV + patients. Genomic DNA was extracted from CD14 + monocytes from NI individuals and untreated HIV + patients categorized as HIV sCD163-low or -high, as an indicator of progression. Determination of the overall DNA methylation was performed using the Imprinting Methylated DNA Quantification kit (Sigma Aldrich). Data is expressed as the mean of the percentage relative to 100% methylated control. *p < 0.05 versus NI.

**Figure 6:**
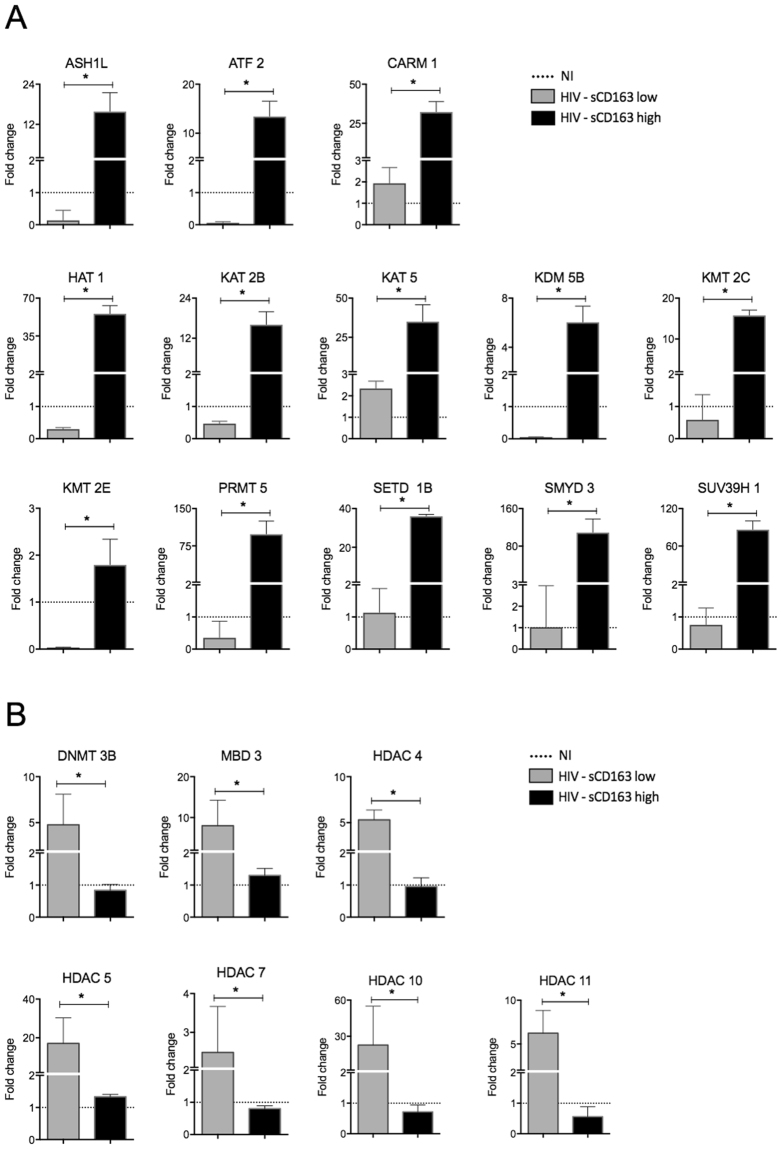
Gene expression of enzymes responsible for epigenetic activation or repression of gene transcription segregated according to the levels of sCD163. Gene expression analysis was performed on CD14 + monocytes from NI individuals and untreated HIV + patients categorized as HIV sCD163-low or -high, as an indicator of progression. PCR Array kit “RT^2^ Profiler^TM^ PCR Array customized by Qiagen (Qiagen/SA Biosciences) was used to assess activation enzymes and TaqMan® Array, Human DNA Methylation & Transcriptional Repression (Applied Biosystems) was used to assess repression enzymes. Data is expressed as fold change relative to the geometric mean of NI. *p < 0.05.

**Figure 7:**
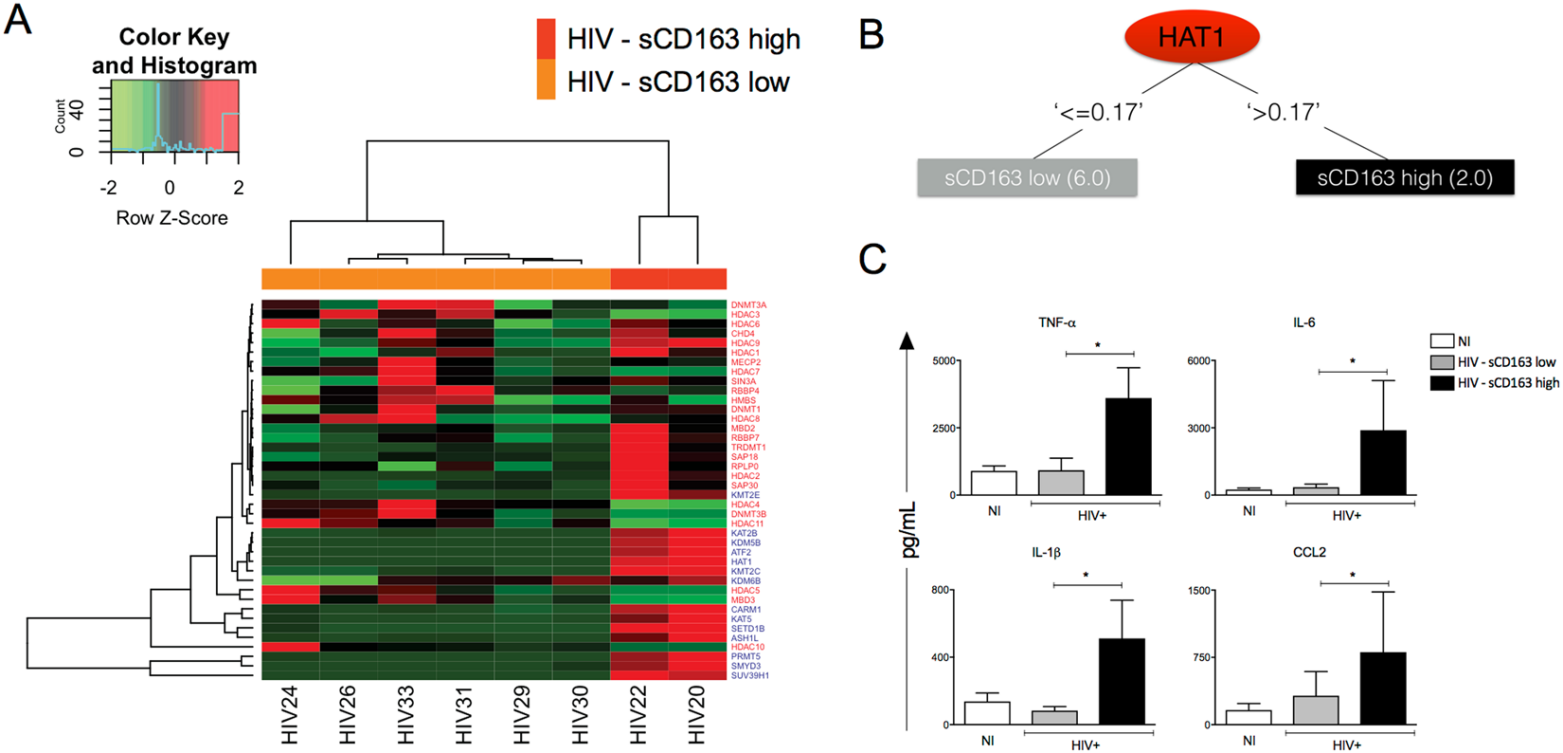
Soluble CD163 levels of untreated HIV + patients are reflected on gene expression of enzymes responsible for epigenetic changes and on functional features of monocytes. (**A**) Gene expression of enzymes responsible for activation or repression of transcription were used to calculate the Z-score for each gene produced by subjects individually from HIV sCD163-low and -high, as an indicator of progression, for further generation of a heat map. Row Z-score scaled from −2 to + 2 and is illustrated as green, black, and red colors. (**B**) The values of the gene expression were used for the decision tree dataset construction. The decision tree was generated considering the two groups separately. The numbers beside the group names indicate the quantity of subjects ranked on each pathway. (**C**) Cytokines and chemokines produced by monocytes from NI individuals, untreated HIV + patients segregated according to the levels of sCD163. Cells were maintained in culture for 24 hours after Mtb infection (MOI 5) and monocyte supernatants were evaluated for the presence of TNF-α, IL-6, IL-1β and CCL2 using magnetic beads from MILLIPLEX MAP kits® Human Cytokine/Chemokine (Millipore). The result is expressed as mean ± SEM. *p < 0.05.

Once segregated according to the levels of sCD163, monocytes from sCD163 high-HIV + patients, HIV22 and HIV20, displayed significant reduction on global DNA methylation, suggesting a more activated microenvironment (Fig. 5). To address whether sCD163 high-HIV + patients would show a differential activation profile, we evaluated gene expression of epigenetic enzymes on monocytes from both groups. HIV - sCD163 high monocytes showed notorious upregulation of all the enzymes responsible for activating gene transcription (Fig. 6A), whereas HIV - sCD163 low exhibited the opposite profile, where enzymes related to gene repression were differentially increased (Fig. 6B). The contrasting patterns on the two subgroups are better visualized on the heat map corresponding to untreated HIV + patients (Fig. 7A) that shows the clear separation regarding epigenetic activation and repression markers according to progression status, considering sCD163 levels. Decision tree pointed out HAT1 as the best epigenetic biomarker on monocytes that differentiate sCD163 high- and low-HIV + patients (Fig. 7B).

### Activated epigenetic biosignature is associated with increased production of inflammatory cytokines on monocytes from sCD163-high HIV patients

To address whether the modulation observed on gene expression profiles of enzymes responsible for epigenetic alterations would be associated with differential cytokines production by the monocytes from sCD163-high HIV patients, cells from NI and untreated HIV + patients were stimulated with Mtb for 24 h and proinflammatory cytokines were assessed. Soluble CD163-high HIV patients showed increased production of TNF-α, IL-6, IL-1β and CCL2 compared to sCD163-low and NI individuals, suggesting that the previous activation state of these cells directly interfere on further dysregulated immune response that contribute to the chronic activation state found in these patients (Fig. 7C).

## Discussion

As it is already well described, progression status in HIV infection is strongly associated with monocyte activation state^2^, but it is necessary to better understand how the virus direct or indirectly is able to modify the function of these cells and how the altered function can affect the course of infection. Monocytes are essential cells in the establishment of innate and adaptive immune responses since they have the ability to differentiate into macrophages and dendritic cells^28^. The differentiation, as well as antigen-presenting ability, migration, chemotaxis, phagocytosis and microbicidal activity reaffirm their crucial role in the pathogenesis of HIV and fighting opportunistic pathogens. We chose to study the functional response of monocytes against *M. tuberculosis* because of the importance of HIV/TB co-infection. The risk of developing tuberculosis (TB) is estimated to be between 26 and 31 times greater in people living with HIV than among those without HIV infection. In 2014, there were 9.6 million new cases of TB, of which 1.2 million were among people living with HIV^29^.

In our study, we show that monocytes from untreated HIV + patients display a complete dysregulated effector function when challenged with Mtb. These cells have reduced ability to phagocytose and kill *M. tuberculosis in vitro* when compared to monocytes from healthy individuals. Several groups before us described impaired phagocytosis from either opsonized^30,31^ or non-opsonized pathogens^32,33^ that are linked to a number of processes in the cells as downregulation of mannose receptor, altered F-actin polymerization and altered signaling pathways^34–36^. The reduction in the microbicidal activity against Mtb in HIV + patients also agrees with previous reports^37–40^. Moreover, our study illustrates the clinical picture of HIV/TB co-infection when we show that among untreated HIV, 64% of the monocytes from these patients were unable to control Mtb growth, while in NI individuals this number is as few as 20% (Fig. 1F). Despite reduced capacity in the elimination and controlling the bacillus, these monocytes showed exacerbated cell activation compared to non-infected individuals, as shown by increased release of inflammatory cytokines, chemokines and reactive oxigen species upon Mtb stimulus compared to NI cells. Thus, despite reduced ability to eliminate and control of the bacillus, these monocytes showed exacerbated cell activation compared to non-infected individuals, which is not correlated to improved microbicidal activity. Furthermore, we detected increased spontaneous production of ROS in monocytes from untreated HIV + patients, once again demonstrating the potential for prior activation of monocytes in these patients (Fig. 2). These findings are in agreement with previous literature^41–44^ and, likewise, correlate with increasing evidence that HIV + individuals suffer immunologic abnormalities similar to non-infected elderly, and thus it is suggested that the progression of the disease is also associated with senescence of the immune system, or a phenomenon called inflammaging^45,46^. Among these signs, the damage caused by mitochondrial dysfunction and increased oxidative stress are determining factors in senescence^47^. Additionally, it has been shown that superoxide, a precursor of free radicals and ROS, regulates major epigenetic processes of DNA methylation, histone methylation and histone acetylation under physiological and pathological conditions^48^. Moreover, ROS-induced oxidative stress is associated with both aberrant hypermethylation of specific promoter regions, but overall global hypomethylation in cancer^49^.

In all the cases above, combining antiretroviral therapy appears to partially restore functional features of the monocytes and reduce intrinsic cell activation, which leads us to some hypothesis. Even though, monocytes are barely infected by HIV, less than 0.1%, viral proteins alone are definitely able to modulate gene expression on monocytes and alter its immune response^43,50–52^. On the other hand, increased chronic cell activation caused by microbial translocation from the gut, may lead to this chronic altered state in the environment orchestrated by epigenetic changes.

For the first time, we are showing that epigenetics plays an important role in monocyte activation from HIV + patients and is directly correlated with disease progression, considering high levels of sCD163 as an indicator of worst prognosis and progression. First, we found that the pattern of global DNA methylation, an alteration responsible for regulating gene transcription was reduced in HIV + individuals (Fig. S1), that is associated with increased activation status of monocytes seen throughout our work. The categorical analysis showed that transcription activation was more evident in the untreated HIV group given the expanded area of the radar charts, showing high frequency of patients with upregulated genes compared to NI individuals. Transcription repression genes were also upregulated, but the overall profile comparing to treated patients is that untreated show more activation marks, while treated show less activation and more repression. Regarding the expression of enzymes responsible for methylation, the balance between downregulation and upregulation of different DNMTs and Methyl-CpG binding proteins could mean an attempt to maintain cellular methylation, moreover, could still be the reason why there is a reduction in global methylation as shown above. Some studies show a correlation between the methylation profile and neurological injuries or cytokines production^53,54^. Furthermore, as an attempt to define the best epigenetic biomarkers to characterize the studied groups we found that HDAC3 and HDAC4 can be used as biomarkers to differentiate untreated from treated patients, as observed in the decision tree (Fig. 4). Regarding the separation of cART patients in two different groups seen in Fig. 4, although we did not explore in the current study, we speculate that the differences observed in the gene expression could be justified by the type of antiretroviral therapy these patients are enrolled. There is a significant IP-10 difference among the two cART groups (data not shown) that agrees with our previous published study showing that patients under second-line therapy have worst prognosis, higher inflammatory profile and higher IP-10 compared to patients enrolled in first-line therapy^27^.

**Figure 8:**
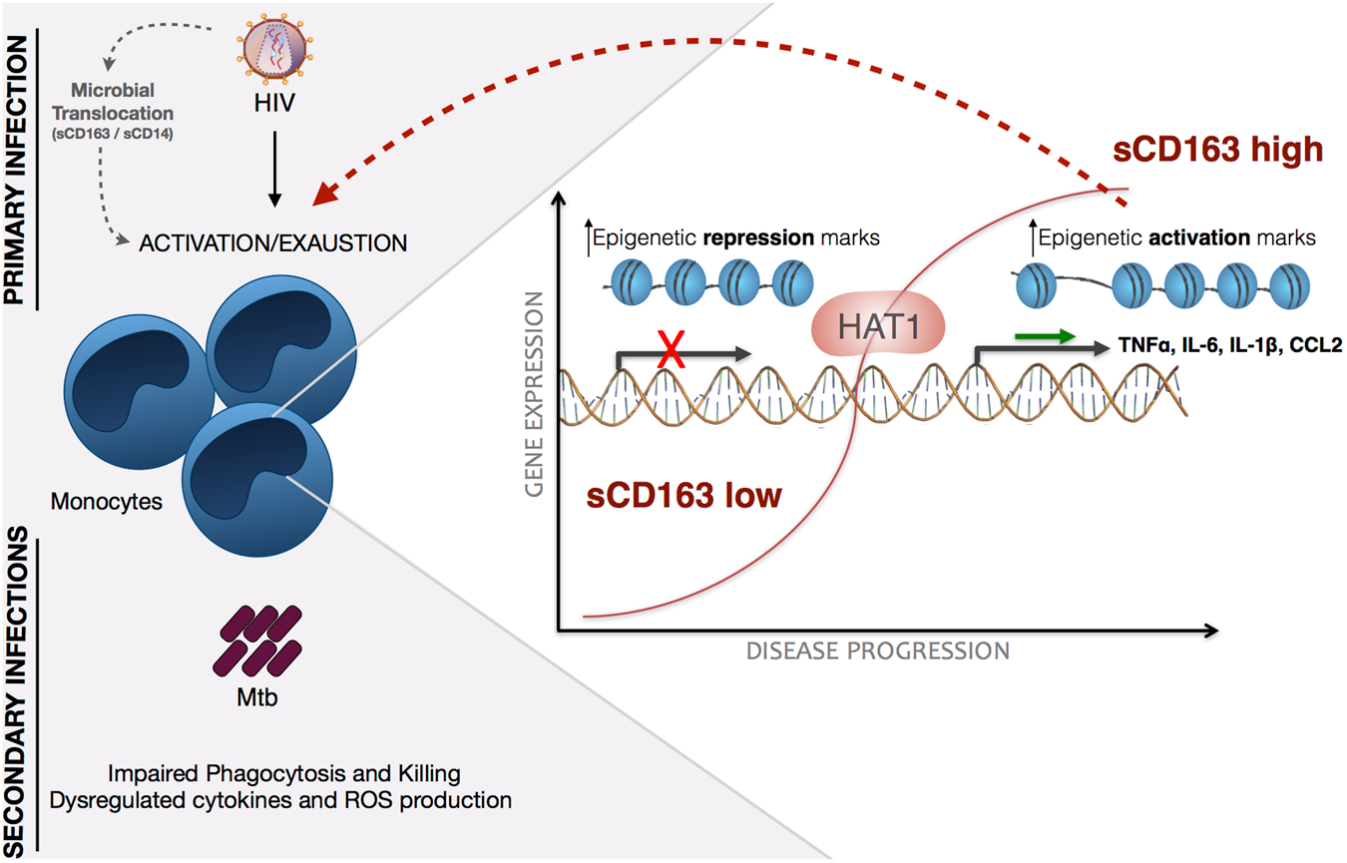
Schematic representation of the events of primary HIV infection and monocyte response in sCD163-low or -high HIV + patients, as an indicator of progression. Upon HIV infection, gut disruption and microbial translocation are some of the most significant events that lead to monocytes chronic activation and exhaustion, quantified by plasmatic levels of sCD163 and sCD14 (not explored in the current work), that directly correlates with systemic immune dysfunction. Here we show that dysregulation of epigenetic events is directly associated with disease progression and proinflammatory cytokines production. The consequences of increased epigenetic activation marks in monocytes may affect its primary functions, as phagocytosis, killing and immune mediators production after a secondary infection, such as *M. tuberculosis*. Histone acetyltransferase 1, HAT1, was the best biomarker that distinguished HIV + sCD163-low and -high patients and is a promising target in therapies aiming reduction of the systemic activation state found in HIV patients.

Once we use “progression” as a factor in HIV-infected untreated patients, here evaluated by plasmatic levels of sCD163 (Fig. S3), we observed an even greater reduction in global DNA methylation of monocytes in sCD163 high HIV + patients and a complete opposite gene expression profile of enzymes responsible for activation and repression of transcription. Soluble CD163 high HIV + patients show increased activation, while patients that have low sCD163 levels show increased repression genes. HAT1 in the case of progression was the best biomarker able to distinguish between sCD163-low and -high HIV + patients (Fig. 8). For the first time its demonstrated that a histone acetyltransferase can be used as a progression biomarker in HIV. Previous studies reported HAT1 being involved in the regulation of HBV replication and that it’s depletion via RNA interference markedly down-regulated virus replication^55^. In esophageal squamous cell carcinoma, HAT1 was an important determinant to regulate the proliferation of human cancerous cells and knockdown of HAT1 induced a G2/M cell cycle arrest, which was associated with the disruption of cell cycle-related events suggesting that HAT1 played an important role in esophageal carcinoma and could potentially be used as a novel therapeutic target^56^.

Thus, in addition to set an epigenetic disease biosignature, we suggest that these enzymes can be used as epigenetic biomarkers associated with treatment and disease progression. Since the expression of enzymes related to activation is associated with an exacerbated cytokine production profile when monocytes are stimulated *in vitro* with Mtb (Fig. 7C), it becomes even more important to use them as therapeutic targets. The expected result would be reduction of the aberrant activation profile of monocytes and its consequences in the immunopathogenesis of the disease, as well as their cellular function. Although there is no sufficient evidence yet to demonstrate the importance of epigenetic regulation of immune cells including monocytes in HIV infection, cancer studies have indicated a clear epigenetic regulation involving cytokines and chemokines, such as TNF-α, IL-1, IL-6 and IFN-γ produced by tumor cells and/or tumor associated leukocytes with the development of malignancies^57^. Recent studies, likewise, include different epigenetic inhibitors as potential treatment of malignancies related to AIDS^58^.

From a clinical point of view, one of the most important characteristics of epigenetic changes is that they can be reversed once using enzymatic treatments, such as HDAC inhibitors and DNMTs, already available on the market. Some of these drugs (5-azacytidine, decitabine, Vorinostat and Romidepsin) have been approved by the FDA for the treatment of hematologic malignancies^59^ and various clinical trials of phase II and III are in development. Although there are no further extensive studies, preliminary data suggest that treatment with epigenetic modifiers in combination with immunosuppressive therapies currently in use, prolongs graft survival in animal models of heart and kidney transplantation^60–62^. In humans, curcumin (a HAT inhibitor) in combination with cyclosporin A can suppress the induction of Th1 lymphocytes in *ex vivo* peripheral blood of transplanted patients^63^.

In conclusion, epigenomics is now a growing area of research in the field of immunology that not only can inspire a new aspect of monitoring immune responses but also open the way for potential treatment strategies in different diseases. In the case of HIV and its immunopathogenesis, in which the state of chronic inflammation remains a determining factor for disease progression, additional studies are needed to qualify the best epigenetic targets and finally validate them as possible weapons against the major deleterious effects of disease.

## Methods

### Ethical Aspects

The study was approved by the Ethics Committee from the Hospital das Clínicas de Ribeirão Preto and FMRP-USP (Protocol #11399/2012). All patients signed an informed written consent form in accordance with the guidelines established by the Brazilian National Health Council. All experiments were performed in accordance with relevant guidelines and regulations.

### Study Population

The selection and contact with patients, collection of cells and clinical follow-up were carried out in collaboration with the Clinical Department UETDI from Hospital das Clínicas de Ribeirão Preto, FMRP-USP, São Paulo, Brazil. Blood samples were collected from untreated HIV-1 + patients (HIV) (n = 17), HIV + patients under antiretroviral therapy for more than six months (HAART) (n = 21) from both sexes and aged between 18 and 65 years. Non-infected individuals (NI) (n = 26) were volunteer healthy blood donors of the Hemotherapy Center of Ribeirão Preto in São Paulo, Brazil, aged between 18 and 65 years, HIV-1 negative and without history of chronic illness or drug use. General characteristics of HIV-1-infected individuals and healthy donors, are listed in Table S1.

### Determination of the concentration of plasma biomarkers

Plasma concentration of sCD163 was measured by ELISA using DuoSet ELISA kit (R&D Systems, Minneapolis, USA) according to the manufacturer’s instructions. CD4 + T-cell counts for HIV-1 + patients were measured by FACS Calibur flow cytometer (Becton-Dickinson, USA). HIV-1 RNA levels in plasma (viremia) were determined by Real Time PCR for HIV-1 (Abbott Molecular).

### Isolation of peripheral blood monocytes

To obtain purified monocytes, blood samples were diluted in phosphate buffered saline (PBS) (1:2) pH 7.4, and PBMC were separated using Ficoll-PaqueTM PLUS, d = 1.078 g/ml (GE Healthcare bio-Sciences AB, Uppsala, Sweden). CD14 + cells were positively selected using immunomagnetic selection with specific kit (Milteny Biotec, Aubum, CA, USA).

### Mycobacterium tuberculosis culture

The Mtb H37Rv (ATCC-27294) strain previously stored at −70 °C were subcultured in liquid medium Midlebrook 7H9 supplemented with 10% oleic acid, albumin, dextrose and catalase (OADC - Difco^TM^, Detroit, USA) and incubated at 37 °C for 11 days until the bacterial growth. Mycobacterial suspension was centrifuged at 2300x g for 20 minutes and the pellet resuspended in sterile glass tube containing saline and sterile glass beads. Bacterial suspensions showing viability higher than 90% were adjusted to 1 × 10^7^ bacilli/ml using nephelometric McFarland scale, then centrifuged at 2300x g for 20 minutes and resuspended in RPMI medium without antibiotic for monocytes stimulation.

### Evaluation of the phagocytic function and microbicide activity of monocytes

To evaluate phagocytosis and microbicidal activity of monocytes, 1 × 10^5^ cells per well were plated in 96 well plates in RPMI 1640 (Gibco, Grand Island, USA) with antibiotics. For each patient, monocytes were plated in two different plates, one for phagocytosis evaluation and the other for microbicide activity. After incubation at 37 °C and 5% CO_2_ overnight for adhesion, the cell supernatant was removed and Mtb suspension was added at a concentration of 5 bacteria/cell (MOI 5). After 2 hours, the timing for phagocytosis evaluation, the supernatant from both phagocytosis and microbicide activity plates were collected and both plates were washed twice with RPMI without antibiotics to remove the bacteria that were not phagocytosed. Then, a saponin solution (0.05%) was added to lyse the cells in the phagocytosis plate and release bacteria in the supernatant for measurement of phagocytic function. Subsequently, resazurin (1 mg/ml) was added to the plate and incubated at 37 °C and 5% CO_2_ for 24 hours. On the second plate, to assess the microbicidal activity after removal of non-phagocytosed bacteria, new RPMI medium was added to the culture, plus 5% FBS and cells were incubated for an additional 22 hours, the time required for microbicidal activity against Mtb by monocytes. At the end of this period, the medium was removed from the microbicide activity plate and saponin solution (0.05%) was added for lysis of monocytes and release of bacteria that remained alive after phagocytosis. Subsequently, resazurin (1 mg/ml) was added and the plate incubated at 37 °C in 5% CO_2_ for 24 hours. The amount of bacteria in either phagocytosis (2 hours after infection) and microbicidal activity (24 hours after infection) timepoints were quantified indirectly by reduction of resazurin in resofurin by viable mycobacteria. The fluorescence of resofurin was read in spectrofluorimeter (Paradigm SpectraMax, Molecular Devices, Sunnyvale, CA, USA) with excitation at 560 nm and emission at 590 nm. The microbicidal activity was obtained by subtracting the fluorescence obtained at 24 h (corresponding to bacteria that survived the microbicidal activity of monocytes) by the fluorescence at 2 h (corresponding to the amount of phagocytosed bacteria).

### Quantification of cytokines in monocyte culture supernatant

In order to determine the concentration of cytokines produced by monocytes after stimulation with Mtb, 1 × 10^5^ cells per well were plated in 96 well plates in RPMI 1640 (Gibco, Grand Island, USA) with antibiotics. After incubation at 37 °C and 5% CO_2_ overnight for adhesion, the cell supernatant was removed and Mtb suspension was added at a concentration of 5 bacteria/cell (MOI 5). After 24 hours of stimulation, supernatant was collected and centrifuged at 2300x g for 20 minutes to remove bacteria. The culture supernatant was stored at −20 °C until the measurement of cytokines. A customized multiplex kit was used to measure TNF-α, IL-6, IL-1β, CCL2, CCL3 and CCL4 according to manufacturer instructions (EMD Millipore Corporation, Billerica, Massachusetts, USA). A fluorescent bead-based instrument (Luminex® MAGPIX® System; Luminex Corporation, Austin, Texas, USA) was used to read each multiplex plate. Luminex bead-based data were analyzed using Milliplex Analyst software v3.5 (Millipore; VigeneTech Inc., Boston, Massachusetts, USA) and a three-parameter logistic curve fit.

### Determination of Reactive Oxygen Species Production by Monocytes

Reactive oxygen species (ROS) produced by previously isolated monocytes, was evaluated by chemiluminescence amplified by luminol. For that, 1 × 10^5^ cells per tube were stimulated with vehicle (spontaneous release), 5 × 10^5^ heat-killed Mtb or Phorbol 12-mystrate 13-acetate (PMA - Sigma-Aldrich, St. Louis, USA) at a concentration of 10^−7^M as positive control. For either treatment luminol was added and chemiluminescence was measured in a kinetics assay that lasted 2 h in a luminometer (LB AutoLumat 953 Multi-Tub Luminometer, Berthold). The data was expressed as area under the curve, calculated using GraphPad Prism 5.0.

### Determination of global methylation of chromosomal DNA

Genomic DNA was extracted from monocytes previously isolated, using the “PureLink Genomic DNA kit” (Life Technologies, New York, USA), according to manufacturer’s instructions. Then, the DNA was quantified in 2.0 Qubit fluorimeter (Life Technologies, New York, USA) and 50 ng of DNA was used for analysis. The profile of relative global methylation was evaluated using the “imprint Methylated DNA Quantification Kit” (Sigma-Aldrich, St. Louis, USA) according to manufacturer’s instructions.

### PCR Array for enzymes that regulate epigenetic changes

Isolated monocytes were used for RNA isolation using TRIzol reagent (Invitrogen, Carlsbad, USA) according to manufacturer’s instructions. The extracted RNA was treated with “Turbo DNase” (Life Technologies, New York, USA) to eliminate any contamination with genomic DNA according to the manufacturer’s instructions and subsequently quantified in Qubit fluorimeter 2.0 and stored at −80 °C. The cDNA was synthesized using the “High Capacity cDNA Reverse Transcription Kit” (Applied Biosystems, Foster City, USA) and subsequently used for quantification of gene expression by quantitative real-time PRC (StepOnePlus Real-Time PCR System, Thermo Fisher Scientific).

In order to identify the gene expression profile of histone modifying enzymes, we used the PCR Array kit for enzymes that induce repression of gene transcription “TaqMan Array Human DNA Methylation & Transcriptional Repression” (Applied Biosystems, Foster City, USA) and the custom array kit for PCR enzymes that promote the activation of gene transcription based on “RT^2^ Profiler^TM^ PCR Array PAHS-085Z” (Qiagen/SA Biosciences, Venlo, Netherlands). In both cases, 30ng of cDNA was used for each tested gene. Table S2 details the list of analyzed genes encoding enzymes responsible for the repression of gene transcription and Table S3 details the list of analyzed genes encoding enzymes responsible for the activation of gene transcription and their constitutive genes, used as endogenous controls (housekeepings). Fold change was calculated using the StepOne Software v2.3.

### Data analysis

This was a descriptive transversal study that applied four data analysis approaches for observational investigation of monocytes functional response and gene expression of epigenetic related enzymes in NI, untreated or treated HIV + patients, as follows: (1) conventional statistical analysis, (2) biomarker signature analysis, (3) heat map group segregation analysis, and (4) decision tree analysis. These approaches have been shown as relevant to detect, with high sensitivity, putative minor changes in the cell profiles that are not detectable by conventional statistical approaches^27,64,65^.

#### Analysis Conventional Statistics

Initially we tested whether the data followed a normal distribution. Considering the nature of the non-parametric set of data, statistical analysis between NI and HIV were performed using the Mann-Whitney test. Further analysis of HIV subgroups was performed using the Kruskal-Wallis test followed by Dunn’s multiple comparison test. Software GraphPad Prism 5.0 (San Diego, CA, USA) was used to calculate statistical analysis and differences were considered significant when P < 0.05.

#### Epigenetic Biosignature Analysis

To assess the epigenetic biomarkers, we used the Fold Change calculation relative to NI. Thus, the relative expression of NI group was normalized to the relative value 1. Patients who showed fold change above 1 were considered *upregulated* and patients who showed fold change below 1 *downregulated*. The upregulated and downregulated genes from each patient were plotted as black and white diagrams in order to calculate the frequency of individuals that show the increased or decreased gene expression in each clinical group. When considering the status of disease progression, we used the progression biomarker sCD163^27^ to classify patients. sCD163 cut-off was calculated as the global median from HIV + groups and patients were classified as “HIV - sCD163 low” or “HIV - sCD163 high” as detailed in Fig. S3. Data are considered significant are when frequency is >50%. Radar charts were constructed to characterize the overall frequency of upregulated genes.

#### Heat Map and Decision Tree

The heat map analysis was carried out using the heatmap.2 function in the R (Project for Statistical Computing Version 3.0.1) and gplots package with the default clustering parameters. All analyses were performed using customized functions available from Bioconductor packages. After dataset analysis, a decision tree was generated for each heat map. The C4.5 algorithm^66^ was used to build the decision tree from WEKA implementation software (Waikato Environment for Knowledge Analysis, version 3.6.11, University of Waikato, New Zealand), using default J48 parameters^67^. The decision trees, the most widely used machine learning algorithms, were used to select the minimal set of phenotypic features that efficiently segregated the groups. This method analyzes all the phenotypic attributes in the training set and selects the most relevant attribute that maximizes the information gain as the root node. The method continues searching for additional attributes for group segregation. To estimate the classification accuracy of the decision tree models on new data with unknown class labels, we used a 10-fold cross validation methodology available in the WEKA software.

### Data availability

The datasets generated during and/or analysed during the current study are available from the corresponding author on reasonable request.

## Electronic supplementary material

Supplementary Information
