# Supplementary material for: Epigenetic alterations are associated with monocyte immune dysfunctions in HIV-1 infection
Source: Sci Rep. 2018 Apr 3;8:5505. doi: 10.1038/s41598-018-23841-1 (PMC5882962; doi:10.1038/s41598-018-23841-1)
Supplement: Supplementary file 1 — Supplementary Information [file 41598_2018_23841_MOESM1_ESM.doc]

**Epigenetic alterations are associated with monocyte immune dysfunctions in HIV-1 infection**

Milena S Espíndola^1^, Luana S Soares^1^, Leonardo J G Lima^1^, Fabiana A Zambuzi^1^, Maira C Cacemiro^1^, Verônica S Brauer^1^, Cleni M Marzocchi-Machado^1^, Matheus de Souza Gomes^2^, Laurence R Amaral^2^, Olindo A Martins-Filho^3^, Valdes R Bollela^4^, Fabiani G Frantz^1^

**1** Faculdade de Ciencias Farmaceuticas de Ribeirao Preto, Universidade de Sao Paulo, Ribeirao Preto, SP, Brazil, **2** Laboratorio de Bioinformatica e Analises Moleculares – INGEB / FACOM, Universidade Federal de Uberlandia, Patos de Minas, MG, Brazil, **3** Laboratorio de Biomarcadores para Diagnostico e Monitoramento, Centro de Pesquisas Rene Rachou, FIOCRUZ, Belo Horizonte, MG, Brazil, **4** Faculdade de Medicina de Ribeirao Preto, Universidade de Sao Paulo, Ribeirao Preto, SP, Brazil

Corresponding author: Fabiani Gai Frantz ([frantz@usp.br)](mailto:frantz@usp.br))

Departamento de Análises Clinicas, Toxicologicas e Bromatologicas, Faculdade de Ciencias Farmaceuticas de Ribeirao Preto, Universidade de Sao Paulo, Av. do Cafe s/n, 14040-903, Ribeirao Preto, Sao Paulo, Brazil

**S1 Table.**  Basic characteristics of untreated or cART treated HIV-1-infected individuals and healthy donors.

| **Baseline Characteristics** | **Values** | | | |
| --- | --- | --- | --- | --- |
|  | **Healthy controls (NI)** | **HIV** | | |
|  |  | **Untreated** | **cART** |  |
| Individuals (n) | 26 | 17 | 21 |  |
| Female [no. (%)] | 11 (42) | 5 (29) | 9 (43) |  |
| Male [no. (%)] | 15 (58) | 12 (71) | 12 (57) |  |
| Age [years (IR)] | 35 (26-46) | 39 (31-47) | 43 (36-53) |  |
| CD4/mL (IR) | NA | 533 (323-692) | 776 (495-978) |  |
| HIV RNA/mL  [log (IR)] | NA | 4,225  (3,47-4,90) | <50 |  |

Numbers are expressed as Mean (%) or Mean (interquartile range), as indicated. IR: interquartile range.

**S2 Table:** List of analyzed genes that encode enzymes responsible for the repression of gene transcription

| **Genes** | | | | | | |
| --- | --- | --- | --- | --- | --- | --- |
| **Target genes** | | | | | | **Endogenous Controls** |
| DNMT1 | MBD3 | HDAC4 | HDAC9 | SIN3A | RBBP7 | GAPDH |
| DNMT3A | MECP2 | HDAC5 | HDAC10 | RPLP0 |  | HPRT1 |
| DNMT3B | HDAC1 | HDAC6 | HDAC11 | HMBS |  | B2M |
| TRDMT1 | HDAC2 | HDAC7 | SAP18 | CHD4 |  | ACTB |
| MBD2 | HDAC3 | HDAC8 | SAP30 | RBBP4 |  | GUSB |

**S3 Table:** List of analyzed genes that encode enzymes responsible for the activation of gene transcription

| **Genes** | | | | | |
| --- | --- | --- | --- | --- | --- |
| **Target genes** | | | | | **Endogenous Controls** |
| ASH1L | HAT1 | KDM5B | KMT2E | SMYD3 | GAPDH |
| ATF2 | KAT2B | KDM6B | PRMT5 | SUV39H1 | ACTB |
| CARM1 | KAT5 | KMT2C | SETD1B |  |  |

**S1 Figure**

**S1 Figure. Percentage of monocytes global methylation of genomic DNA.** Genomic DNA was extracted from CD14+ monocytes from NI, untreated HIV+ patients and HIV+ patients under HAART. Determination of the overall DNA methylation was performed using the Imprinting Methylated DNA Quantification kit (Sigma Aldrich). Data is expressed as the mean of the percentage relative to 100% methylated control. *p<0,05 versus NI; # p<0,05 versus HIV.

**S2 Figure**

**S2 Figure. Correlation analysis between sCD163 levels and the clinical outcomes CD4 T cell counts, CD4/CD8 ratio, Viremia and Plasmatic IP-10 in HIV+ patients.** Plasma was separated and subsequently used for soluble CD163 measurement by ELISA. CD4 T-cell counts were measured by flow cytometry. HIV-1 RNA levels in plasma (viremia) were determined by Real Time PCR for HIV-1.

**S3 Figure**

**S3 Figure. Plasma levels of soluble CD163 for segregation between sCD163 low and sCD163 high**. Plasma was separated and subsequently used for soluble CD163 measurement by ELISA. The median from HIV group was used as the cut-off edge between sCD163 low and high. *P <0.05 versus NI; # P <0.05 versus HIV.
